# CT Imaging of Craniofacial Fibrous Dysplasia

**DOI:** 10.1155/2015/134123

**Published:** 2015-08-03

**Authors:** Zerrin Unal Erzurumlu, Peruze Celenk, Emel Bulut, Yakup Sancar Barıs

**Affiliations:** ^1^Department of Oral and Maxillofacial Radiology, Ordu University, Faculty of Dentistry, 52100 Ordu, Turkey; ^2^Department of Oral and Maxillofacial Radiology, Ondokuz Mayis University, Faculty of Dentistry, 55100 Samsun, Turkey; ^3^Department of Oral and Maxillofacial Surgery, Ondokuz Mayis University, Faculty of Dentistry, 55100 Samsun, Turkey; ^4^Department of Pathology, Ondokuz Mayis University, Faculty of Medicine, 55100 Samsun, Turkey

## Abstract

Fibrous dysplasia is a benign fibroosseous bone dysplasia that can involve single (monostotic) or multiple (polyostotic) bones. Monostotic form is more frequent in the jaws. It is termed as craniofacial fibrous dysplasia, when it involves, though rarely, adjacent craniofacial bones. A 16-year-old girl consulted for a painless swelling in the right posterior mandible for two years. Panoramic radiography revealed ground-glass ill-defined lesions in the three different regions of the maxilla and mandible. Axial CT scan (bone window) showed multiple lesions involving skull base and facial bones. Despite lesions in the skull base, the patient had no abnormal neurological findings. The lesion was diagnosed as fibrous dysplasia based on radiological and histopathological examination. In this paper, CT findings and differential diagnosis of CFD are discussed. CT is a useful imaging technique for CFD cases.

## 1. Introduction

Fibrous dysplasia is a developmental tumor-like disease having characteristics of replacement of normal bone by an excessively proliferated cellular fibrous connective tissue intermixed with irregular bony trabeculae [1]. Fibrous dysplasia (FD) may affect single (monostotic = MFD) or multiple (polyostotic = PFD) bones. Monostotic FD is called CFD when it occurs adjacent to the craniofacial bones. However, CFD is a rare form of FD. Craniofacial involvement in CFD most commonly results in neurological symptoms like hearing loss, visual loss, headache, proptosis, and so forth [1, 2].

The case presented here was incidentally revealed during the intraoral examination of the patient. Radiological examination showed that several craniofacial bones were affected. CFD was diagnosed with both CT and histopathologic examination.

## 2. Case Report

**Figure 1:**
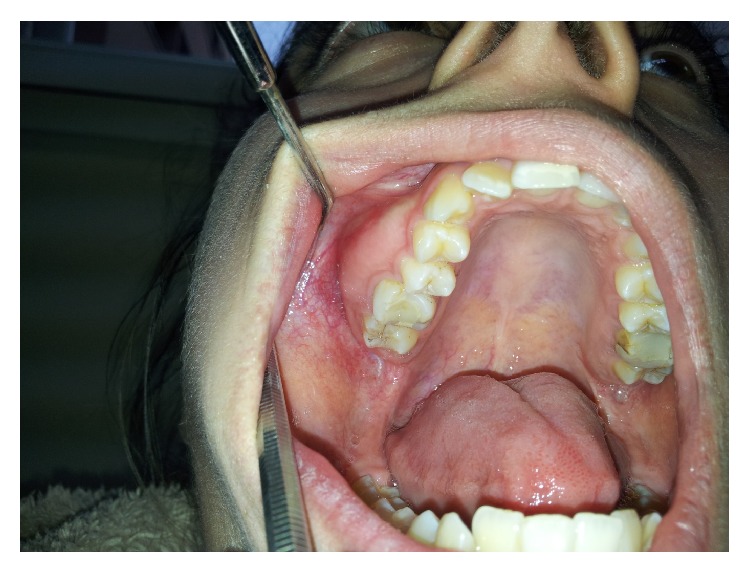
Expansive mass of the right maxilla.

A 16-year old girl complaining of malocclusion was presented. Her medical history was unremarkable. Intraoral examination revealed an expansion in the buccal direction at the right maxilla. The overlying mucosa had normal color and appearance (Figure 1).

**Figure 2:**
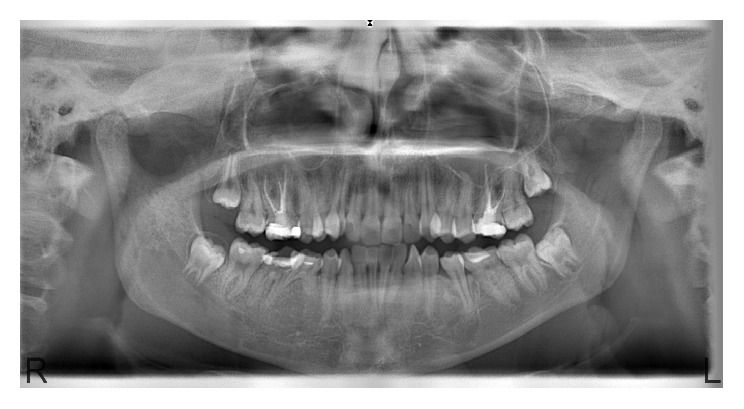
Panoramic radiography shows ground-glass ill-defined lesions in the three different regions of the maxilla and mandible.

Panoramic radiograph showed ground-glass ill-defined lesions in right mandibular corpus and ramus, right maxillary posterior region, and mandibular symphysis region (Figure 2).

**Figure 3:**
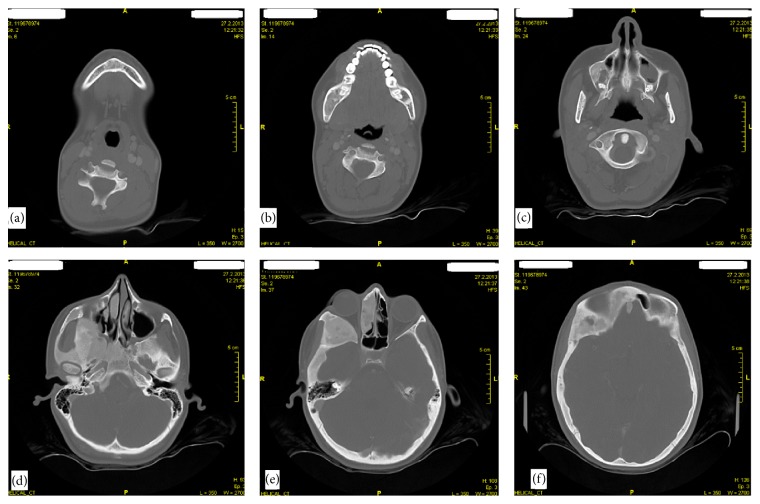
(a), (b), (c), (d), (e), (f) Axial CT scans (bone window) showing multiple bone lesions involving skull base and facial bones.

**Figure 4:**
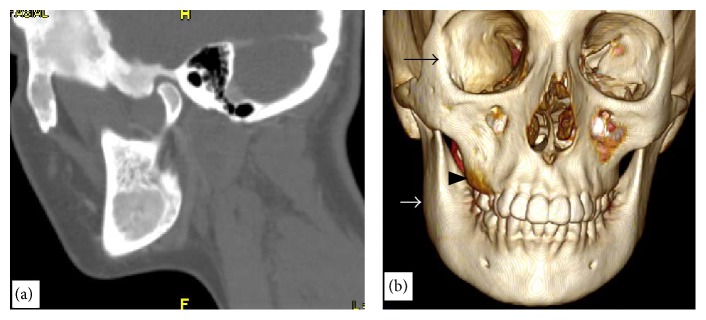
(a) Sagittal reformatted CT images showing expansive mixed lesions involving the mandible, temporal bone, and zygomatic bone. (b) 3D CT images showing the CFD affecting the right side of the mandible (white arrow), in the right maxilla (black arrow head), in the right lateral wall of the orbit (black arrow).

CT imaging showed expansive mass with ground-glass opacity involving the mandible, maxilla, sphenoid, frontal, ethmoid, zygomatic, temporal bones, and clivus (Figures 3 and 4).

Skeletal survey did not show any involvement of other bones in the skeleton. The patient's blood glucose, serum calcium, phosphate, alkaline phosphate, and parathormone levels were within normal limits.

**Figure 5:**
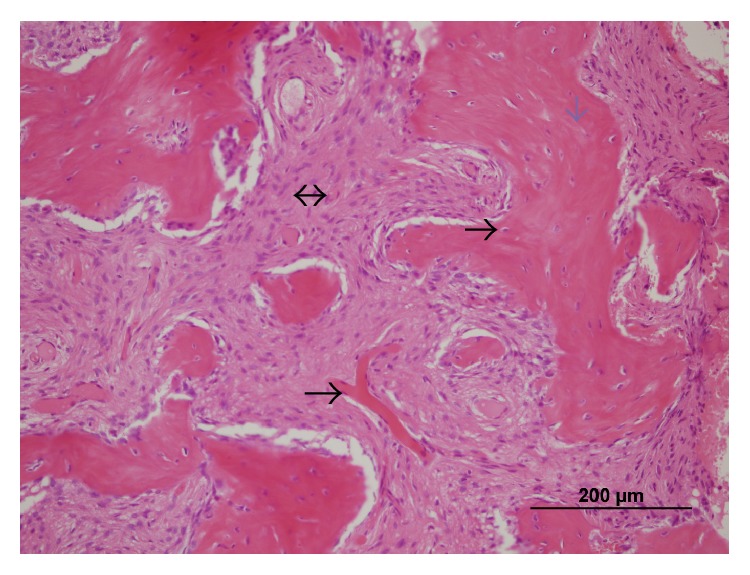
Histopathology demonstrates immature bone anastomosis, forming at the fibrous stroma rich of cellular and vascular structure. Vascular structure (black arrow), osteocyte (small black arrow), osteoid matrix (blue arrow), and fibrous stroma (two-sided arrow) are shown (HE ×200).

Incisional biopsy was performed on the right mandibular lesion under local anesthesia. Histopathology revealed that the tumor was composed of a solid proliferation of spindle-shaped cells associated with islands of osteoid and bone trabeculae. The trabeculae of woven bones had irregular size, form, and distribution (Figure 5). Based on clinical, radiological, and histopathological examinations, the lesions were diagnosed as craniofacial fibrous dysplasia (CFD).

## 3. Discussion

Fibrous dysplasia (FD) is a sporadic genetic disease of bone that may affect single or multiple bones. When the disease is limited to a single bone, it is termed as monostotic fibrous dysplasia. Although mandibular lesions are truly monostotic, maxillary lesions often involving adjacent bones, such as zygomatic, sphenoid, and occipital, are not strictly monostotic. The designation of CFD was appropriate for these lesions [1]. In the present case, CT images showed expansive mass at ground-glass density that involved mandibula, maxilla, sphenoid, frontal, ethmoid, zygomatic, temporal bones, and clivus.

FD can be seen in children and young adults. The monostotic form (MFD) is equally distributed in both genders and is six times more common than the polyostotic one. In the monostotic form, maxilla is involved more than mandible (max/mand = 2/1), and it occurs unilaterally at the posterior of the jaws [1–4].

Three different radiographical patterns have been defined for FD. These are cystic (radiolucent or lytic), sclerotic, and mixed (radiolucent/radiopaque) [1, 2].

Jaw lesions cause displacement of teeth, loss of lamina dura, narrowing of the periodontal ligament space, and rarely root resorption [1, 3]. Superior displacement of the mandibular canal is suggestive of FD [2]. Nasal obstruction may occur if paranasal sinuses are affected. Lesions extending to the orbit may cause visual impairment and temporal bone lesions may cause hearing loss. Facial pain, headaches, or facial numbness may develop [3, 5].

Asymmetric homogeneous “ground-glass” appearances that blend into normal bone, thin cortices, and bone expansion are the main characteristics for FD [2]. Panoramic radiographs showed involvement and extension of the jaws well. CT (bone algorithm) and CBCT are useful in the determination of craniofacial involvement and to check neural and vascular foramen [2, 3, 6].

Differential diagnosis is made through Paget's disease, osteomyelitis, osteosarcoma, and cementoossifying fibroma [2]. Paget's disease affects older age group and causes enlargement of the entire jaw. Osteomyelitis may cause jaw enlargement from periosteum having sequester. Osteosarcoma may have characteristic periosteal response. Ossifying fibroma is generally a well-defined lesion with a smooth margin and has tumor-like concentric expansion [2].

Typical microscopic finding of FD is immature bone that contains irregularly shaped trabeculae within the cellular, loosely organized fibrosis stroma [1].

In most cases of FD, the lesions seem to be stabilized with skeletal maturation. Recurrence or reactivation has been reported in 18% of the cases. Simple bone cyst, central giant cell granuloma, or aneurysmal bone cyst may develop in the affected bone [2]. If there is any occurrence of aesthetical defects, functional or aesthetic surgical interventions may be necessary. FD lesions when subjected to X-rays may show malignant transformation [1].

In conclusion CT is a useful imaging technique in CFD cases for showing the involvement of facial and skull base bones, evaluation of neural and vascular foramen, and follow-up of the patients.
